# Analysis of naltrexone and its metabolite 6-beta-naltrexol in serum with high-performance liquid chromatography

**DOI:** 10.1186/1756-0500-5-439

**Published:** 2012-08-15

**Authors:** Pekka Heinälä, Tuuli Lahti, David Sinclair, Kari Ariniemi, Pirjo Lillsunde, Hannu Alho

**Affiliations:** 1 National Public Health Institute, Helsinki, Finland; 2 Finnish Foundation for Alcohol Studies, Helsinki, Finland; 3 Department of Behavioural Sciences and Philosophy, University of Turku, Turku, Finland; 4 Research Unit of Substance Abuse Medicine, University of Helsinki, Helsinki, Finland; 5 THL, PL 30, 00271, Helsinki, Finland

**Keywords:** Naltrexone, 6-β-naltrexol, High-performance liquid chromatography, Alcoholism

## Abstract

**Background:**

Naltrexone has been proven to be an effective treatment option for the treatment of alcohol dependency. In this article we introduce a reliable and simple method developed for the simultaneous determination of naltrexone and 6-β-naltrexol in human serum by using high-performance liquid chromatography (HPLC).

**Findings:**

Liquid-liquid extraction with butyl acetate from basic solutions (pH 9) was chosen for extraction with nalorphine as an internal standard (IS). Analytes were back-extracted from organic solvent into perchloric acid. The acid extract was chromatographed by HPLC with a reverse-phase ODS-column and electrochemical detector. The mobile phase was a NaH_2_PO_4_-solution with acetonitrile as an organic modifier and octanesulphonic acid and tetraethylammonium hydrogen sulphate as ion-pair reagents. The recovery of the extraction method was 48% for naltrexone and 75% for 6-β-naltrexol. The limit of quantification was 5.0 ng/ml for naltrexone and 1.0 ng/ml for 6-β-naltrexol. The analysed concentrations of naltrexone differed from the theoretic concentrations by 0.7 to 2.3% and those of 6-β-naltrexol by 2.6%. The relative standard deviation of within-day assay was from 0.9 to 5.7% for naltrexone and from 0.8 to 4.2% for 6-β-naltrexol; for the between-day assay it was 5.7% and 4.2%, respectively.

**Conclusions:**

Our results indicate that the developed method is suitable for determination of naltrexone and 6-β-naltrexol in human serum.

## Findings

**Figure 1:**
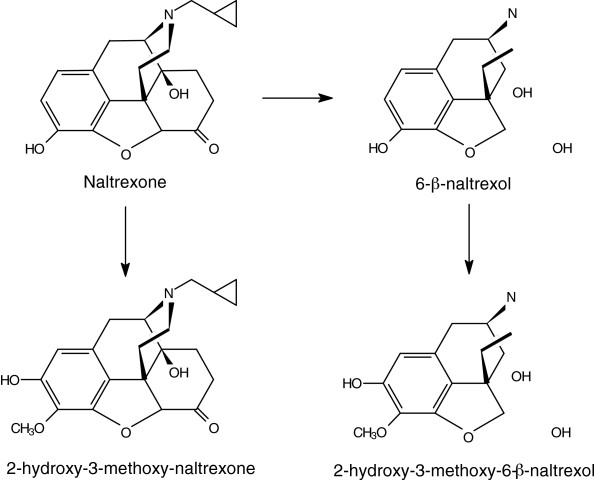
Metabolism of Naltrexone.

Naltrexone is an opioid receptor antagonist, which has been used decades for the treatment of alcoholism [1-4] and opiate dependency [5-7]. Several well-controlled clinical trials have demonstrated that naltrexone is an efficacious adjunctive medication in the treatment of alcoholism [2,8,9]. Naltrexone has shown to reduce relapse rates and alcohol craving among alcoholics as compared to placebo-treated individuals [2]. Once administered, naltrexone is known to undergo a rapid and extensive hepatic metabolism by enzymatic reduction from ketone to a major metabolite 6-β-naltrexol (Figure 1) and to other minor metabolites. 6-β-naltrexol is weaker opioid receptor antagonist than naltrexone, but it may contribute to the clinical effects of the drug as it persists in biological fluids in higher amounts than naltrexone [10]. Naltrexone and its metabolites are mostly present in conjugated forms [11-13]. Less reduction of naltrexone to 6-β-naltrexol seems to occur in liver cirrhosis, and such alterations appear to be related to the severity of liver disease [14,15]. 

Several methods for therapeutic drug monitoring of naltrexone and 6-β-naltrexol have been developed over the years. These include gas chromatography with electron capture detection (GC-ECD) [13,16], high-performance liquid chromatography with electrochemical detection (HPLC-EC) [17,18], gas chromatography–mass spectro-metry (GC-MS) [19,20], and gas chromatography-tandem mass spectrometry (GC-MS-MS) [21]. Recently Brünen et al. [22] introduced two novel methods, HPLC/UV (high-performance liquid chromatography and UV spectrophotometric detection) and LC-MS/MS (liquid-chromatographic method coupled with tandem mass spectrometry), for the determination of naltrexone and 6-β-naltrexol. The methods developed by Brünen et al. [22] have several benefits, such as better automatization and higher sensitivity, as compared to other methods developed for the simultaneous quantitative determination of naltrexone and 6-β-naltrexol in biological fluids.

With therapeutic drug monitoring the treatment outcomes of drugs can be significantly improved. It has been suggested that therapeutic drug monitoring should be a standard protocol while using naltrexone for the treatment of substance abuse [23]. Thus we aimed to develop even more efficient analysis method for determi-nation of naltrexone and 6-β-naltrexol in human serum than has been earlier developed. To achieve this goal we used liquid-liquid extraction and subsequent analysis by HPLC with electroche-mical detection for determination of naltrexone and 6-β-naltrexol in human serum.

### Materials and methods

The naltrexone (Dupont Merck) and 6-β-naltrexol (NIDA) stock solutions were prepared in methanol to give concentrations of 1 mg/ml of free base. These solutions were used to prepare calibration curves covering the range from 0 to 1000 ng/ml. All calibration standards, blanks and quality control samples, were prepared in drug-free cattle serum. All buffers were prepared with deionized water.

### Clinical sampling

Samples from 87 (23 women and 64 men) alcoholics (DSM-IV) were taken. The alcoholics were participating in a double blind, placebo-controlled, prospective clinical trial evaluating naltrexone in the treatment of alcohol dependence. The main inclusion criteria were: age between 18–65, alcohol dependency (DSM-IV and ICD-10). Naltrexone (naltrexone HCl, ReVia®) was taken daily 50 mg orally every morning for a three-month period. The blood sample was collected approximately 2–4 h after the medication consumption. Fasting serum samples were collected by vein puncture at weeks 0, 2, and 8, and allowed to clot, and centrifuged (1000 g, 10 min, at room temperature). All samples were stored at −70°C until analysis.

### Extraction

1 ml of serum was extracted for all standard, quality control, and unknown clinical samples. The pH of the sample was adjusted to pH 9 by 0.5 M Na_2_HPO_4_⋅2H_2_O. The sample was extracted with 5 ml of butyl acetate with nalorphine (1 μg / 100 ml) as the internal standard. After vortex-mixing (30 s), the sample was centrifuged, and the upper butyl acetate layer was transferred to a clean test tube and back-extracted into 150 μl of 0.1 M HClO_4_. After vortex-mixing (30 s) and centrifugation, the upper butyl acetate phase was thrown out. The acid phase was transferred to the autosampler vial, and 25 μl of the acid phase was injected into the HPLC.

### Chromatographic conditions

A Hewlett-Packard 1090 Series II high-performance liquid chromatography with an Esa Coulochem 5100A electrochemical coulometric detector (potentials: Detector 1 + 0.2 V, Detector 2 + 0.5 V) was used. The mobile phase was acetonitrile-potassium dihydrogen phosphate (19 mM) (10:45, v/v) with 1-octanesulphonic acid (5 mM) and tetraethylammonium hydrogen sulphate (5 mM) as ion-pair reagents. Chromatographic sepa-ration was achieved using an ODS Hypersil reverse-phase column (Hewlett Packard) with a length of 125 mm, an i.d. of 4 mm, and a particle size of 5 μm. The mobile phase was pumped at a flow-rate of 1.2 ml/min.

The identification of drugs was based on retention times, which were checked with calibration standards before each run. An internal standard method with linear one-point calibration based on peak heights was used for quantification. Reliability of the method was tested for linearity, accuracy, and within-day and between-day assay precision. The suitability for determination of naltrexone and its major metabolite was tested by analysing serum samples from patients participated in alcoholism treatment with naltrexone.

### Results and discussion

**Figure 2:**
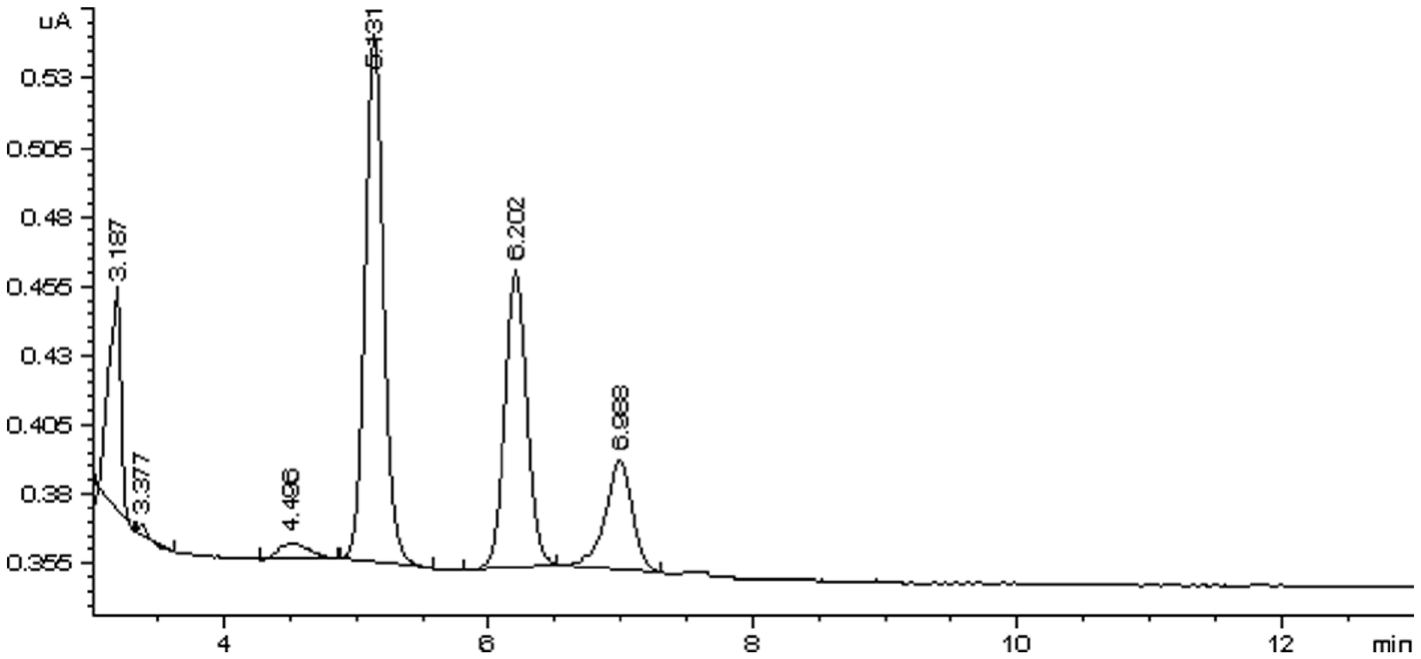
A chromatogram of a serum standard containing 50 ng/ml naltrexone (RT = 6.99) and 6-β- naltrexol (RT = 6.20), nalorphine (RT = 5.13) as internal standard.

**Figure 3:**
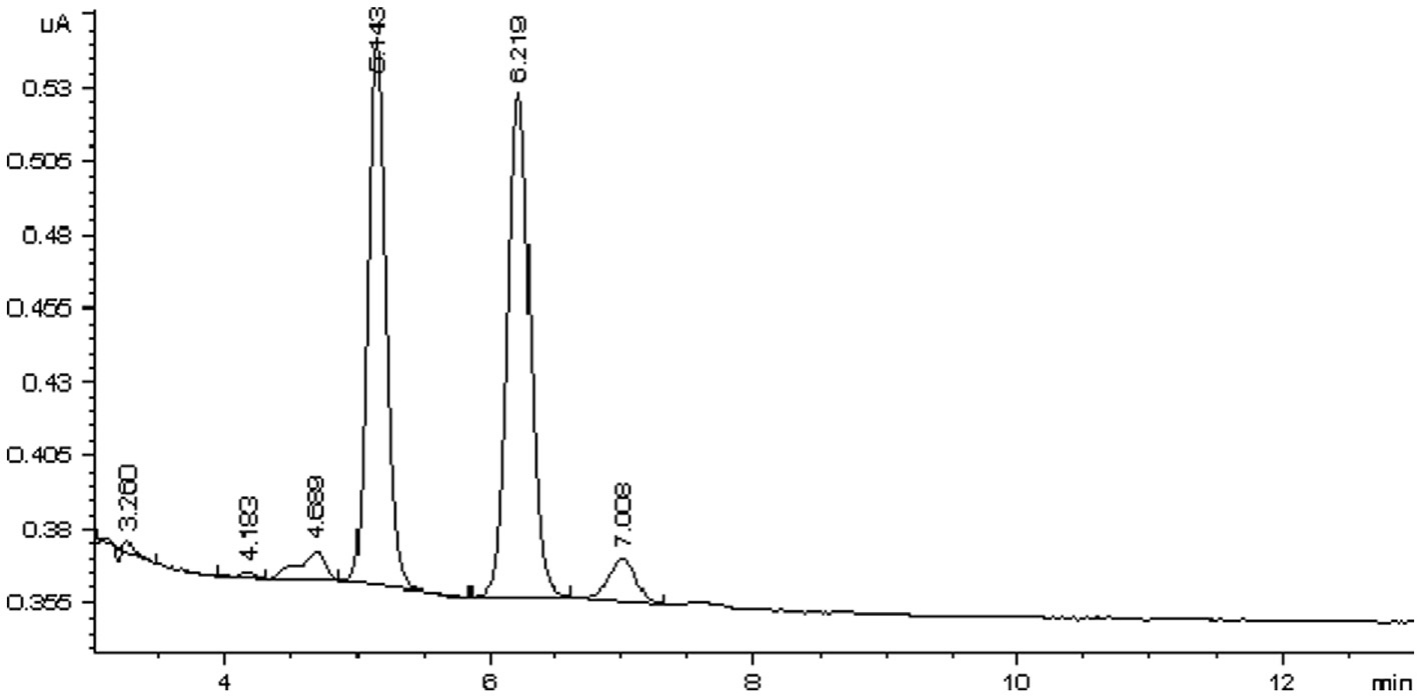
A chromatogram of a patient sample containing naltrexone (RT = 7.00) and 6-β-naltrexol (6.22), nalorphine as internal standard (RT = 5.14).

Chromatograms of the control samples spiked with naltrexone and 6-β-naltrexol and an unknown clinical serum sample are all shown in Figures 2 and 3. The retention times (min) of nalorphine (IS), 6-β-naltrexol, and naltrexone were 5.1, 6.1, and 6.9, respectively. The extraction recoveries at three concentration levels (100, 250, and 500 ng/ml) varied from 46.5 to 50.7% for naltrexone and from 71.6 to 77.4% for 6-β-naltrexol. Two sets of standard samples were prepared at a concentration range from 0 to 1000 ng/ml to determine the linearity. The linearity ranges and the mean correlation coefficients (r^2^) for naltrexone and 6-β-naltrexol are shown in Table 1. The method was linear wide over the concentration range of naltrexone and 6-β-naltrexol in clinical samples.

**Table 1:** **The linearity range, mean correlation coefficient (r**^**2**^**), and limit of quantification(LQ) with RSD-value for naltrexone and 6-β-naltrexol**

**Compound**	**Linearity range (ng/ml)**	**r**^**2**^	**LQ (ng/ml)**	**RSD (%)**
Naltrexone	5-1000	0.9987	5	5.7
6-β-naltrexol	1-1000	0.992	1	15.4

The limit of quantification was determined by analysing sets of six samples at low concentration levels. Relative standard deviations (RSD) of these samples were calculated. Signal-to-noise ratio of at least three and RSD-values below 20% were used as criteria for the limit of quantification. The limits of quantification with RSD-values for both compounds are shown in Table 1. The accuracy and within-day precision of the assay was tested at two concentration levels and with six samples. The average of concentrations found, the accuracy, and the within-day assay precision for naltrexone and 6-β-naltrexol are shown in Table 2.

**Table 2:** Average concentrations found, accuracy and within-day assay precision for naltrexone and 6-β-naltrexol

**Compound**	**Concentration added (ng/ml)**	**Concentration found (ng/ml)**	**Accuracy (%)**	**Precision (%)**
Naltrexone	5	4.97	0.7	5.7
	150	146.6	2.3	0.9
6-β-naltrexol	5	4.87	2.6	4.2
	150	146.1	2.6	0.8

Between-day precision of the assay was determined by analysing the same serum standard 15 times in 30 days. A serum standard, spiked with 100 ng/ml of naltrexone and 6-β-naltrexol, was stored at −20°C. The average concentrations found were 98.8 and 98.9 ng/ml, and between-day assay precision was 5.7% and 4.2% for naltrexone and 6-β-naltrexol, respectively. Small changes in laboratory conditions (room temperature, atmospheric moisture) had no effect on the results. Retention times seemed to increase slightly because of contamination within the chromatographic system. This had, however, no effect on results because the retention times were checked with calibration standards before each determination. Although the extraction recovery was relatively low, the accuracy and the precision were good. The specific stability validation was not performed, but the between-day precision showed that the serum standard samples were stable for 30 days at −20°C. The clinical serum samples were stored at −70°C.

On the basis of validation results, the developed method was found suitable for determination of naltrexone and 6-β-naltrexol in human serum. The within-day and between-day assay precision was acceptable for all tested concentration levels. The developed method was used for determination of naltrexone and 6-β-naltrexol in serum samples from patients participating in naltrexone treatment of alcoholism. Analysed serum samples were collected after two and eight weeks of continuous naltrexone treatment. Concentrations of naltrexone and its major metabolite in serum samples varied greatly between patients. Naltrexone concentrations in serum were 0–70 ng/ml after two weeks and 0–19 ng/ml after eight weeks of continuous naltrexone treatment. 6-β-naltrexol concentrations were 15–136 ng/ml and 3–163 ng/ml after two and eight weeks, respectively. 6-β-naltrexol seemed to be the more reliable compound to monitor because some unidentified compound was found to interfere with the quantification of naltrexone. The laboratory verification demonstrated the tests were accurate in showing the levels of naltrexone and naltrexol in serum samples, but high variability was seen in the results from patients reporting that they were taking naltrexone. The reasons for the variability in practice remain to be determined but could have an influence on the efficacy of the treatment.

## Abbreviations

HPLC: High-performance liquid chromatography; IS: Internal standard; GC-ECD: Gas chromatography with electron capture detection; HPLC-EC: High-performance liquid chromatography with electrochemical detection; GC-MS: Gas chromatography-mass spectrometry; GC-MS-MS: Gas chromatography-tandem mass spectrometry; RSD: Relative standard deviations.

## Competing interests

The authors declare that they have no competing interests.

## Authors’ contributions

PH participated in the design of the study, made substantial contributions to the analysis of the data and drafted the manuscript. TL and DS were involved in drafting and revising the manuscript and in the interpretation of the data. KA and PL conceived of the study, and participated in its design and coordination. HA participated to the design and acquisition of the data as well as to the interpretation of the data. All authors read and approved the final manuscript.
